# Differences in Beef Quality between Angus (*Bos taurus taurus*) and Nellore (*Bos taurus indicus*) Cattle through a Proteomic and Phosphoproteomic Approach

**DOI:** 10.1371/journal.pone.0170294

**Published:** 2017-01-19

**Authors:** Rafael Torres de Souza Rodrigues, Mario Luiz Chizzotti, Camilo Elber Vital, Maria Cristina Baracat-Pereira, Edvaldo Barros, Karina Costa Busato, Rafael Aparecido Gomes, Márcio Machado Ladeira, Taiane da Silva Martins

**Affiliations:** 1 Department of Animal Science, Universidade Federal de Lavras, Lavras, Minas Gerais, Brazil; 2 Center for Analysis of Biomolecules, Nubiomol, Universidade Federal de Viçosa, Viçosa, Minas Gerais, Brazil; 3 Department of Biochemistry and Molecular Biology, Universidade Federal de Viçosa, Viçosa, Minas Gerais, Brazil; 4 Department of Animal Science, Universidade Federal de Viçosa, Viçosa, Minas Gerais, Brazil; University of Illinois, UNITED STATES

## Abstract

Proteins are the major constituents of muscle and are key molecules regulating the metabolic changes during conversion of muscle to meat. Brazil is one of the largest exporters of beef and most Brazilian cattle are composed by zebu (Nellore) genotype. *Bos indicus* beef is generally leaner and tougher than *Bos taurus* such as Angus. The aim of this study was to compare the muscle proteomic and phosphoproteomic profile of Angus and Nellore. Seven animals of each breed previously subjected the same growth management were confined for 84 days. Proteins were extracted from *Longissimus lumborum* samples collected immediately after slaughter and separated by two-dimensional electrophoresis. Pro-Q Diamond stain was used in phosphoproteomics. Proteins identification was performed using matrix assisted laser desorption/ionization time-of-flight mass spectrometry. Tropomyosin alpha-1 chain, troponin-T, myosin light chain-1 fragment, cytoplasmic malate dehydrogenase, alpha-enolase and 78 kDa glucose-regulated protein were more abundant in Nellore, while myosin light chain 3, prohibitin, mitochondrial stress-70 protein and heat shock 70 kDa protein 6 were more abundant in Angus (*P*<0.05). Nellore had higher phosphorylation of myosin regulatory light chain-2, alpha actin-1, triosephosphate isomerase and 14-3-3 protein epsilon. However, Angus had greater phosphorylation of phosphoglucomutase-1 and troponin-T (*P*<0.05). Therefore, proteins involved in contraction and muscle organization, myofilaments expressed in fast or slow-twitch fibers and heat shock proteins localized in mitochondria or sarcoplasmic reticulum and involved in cell flux of calcium and apoptosis might be associated with differences in beef quality between Angus and Nellore. Furthermore, prohibitin appears to be a potential biomarker of intramuscular fat in cattle. Additionally, differences in phosphorylation of myofilaments and glycolytic enzymes could be involved with differences in muscle contraction force, susceptibility to calpain, apoptosis and postmortem glycolysis, which might also be related to differences in beef quality among Angus and Nellore.

## Introduction

Brazil is the second largest producer and one of the largest beef exporters in the world [1]. The majority of the Brazilian herd is composed of Zebu cattle (*Bos taurus indicus*), mainly the Nellore breed. Zebu beef is usually less tender and has less marbling than that of taurine cattle (*Bos taurus taurus*), particularly the Angus breed [2, 3]. This reduces attractiveness of zebu beef, because tenderness and marbling are considered the main palatability characteristics by consumers [4].

There is an increasing number of studies aiming to understand the molecular mechanisms related to the differences in beef quality between zebu and taurine cattle genotypes [5, 6]. These studies look for biomarkers that might be used in livestock breeding programs. Moreover, they may provide scientific support for the meat industry in the development of strategies to improve meat quality [7, 8, 9].

Proteomics has been widely used for identification of proteins related to meat quality features, because proteins are the major constituent of muscle tissue and also responsible for the regulation of metabolic routes involved in the conversion of muscle to meat [10, 11]. Furthermore, proteomics can be used to study post-translational modifications, which may modify structure and, consequently, protein activity. Phosphorylation stands out among the main post-translational modifications, and phosphoproteomics is a useful technique to study phosphorylated proteins. In muscle tissue, phosphorylation could modulate the interaction among myofilaments and the activity of metabolic enzymes [12, 13, 14].

Proteomic studies comparing fresh muscle or beef from cattle breeds with different beef quality merits were able to identify differentially abundant proteins related to beef sensory attributes [15, 16, 17]. However, proteomics and phosphoproteomics studies comparing muscle or beef of zebu and taurine have not been conducted. Thus, the aim of this study was to compare the muscle proteomic and phosphoproteomic profile of Angus and Nellore cattle.

## Materials and Methods

### Ethical approval

All animal procedures were approved by the Animal Care and Use Committee of the Universidade Federal de Lavras, Brazil, protocol number 048/12.

### Animal handling, slaughter and muscle sampling

Seven Nellore (BW = 375 ± 13 kg) and seven Angus bulls (BW = 383 ± 16 kg), with approximately 20 months of age and previously subjected the same growth management under grazing were confined *ad libitum* for 84 days with a standard feedlot diet used in Brazil based on corn silage and a corn-soybean meal concentrate with a roughage to concentrate ratio of 30:70. The animals were confined in covered individual stalls with concrete floor and equipped with drinking and feeding troughs. Detailed information about the diet and its chemical composition were previously published [6].

The slaughter was preceded by cerebral concussion followed by exsanguination. There was no electrical stimulation of carcasses. Immediately after exsanguinations, samples were collected from the *Longissimus lumborum* muscle between the 12th and 13th ribs, via incision through hide, and frozen in liquid nitrogen. Samples were then pulverized in liquid nitrogen and stored at -80°C until protein extraction.

### Protein extraction and quantification

Approximately 100 mg of frozen muscle was added to a microtube containing 1 mL of extraction solution [(7 M) urea, (2 M) thiourea, (4% w/v) CHAPS, (1% w/v) dithiothreitol, (2% v/v) immobilized pH gradient (IPG) buffer, pH 4 to 7, (0.5 mM) benzamidine hydrochloride hydrate and (0.5 mM) phenylmethanesulfonyl fluoride]. Muscle sample and extraction solution were homogenized using LabGEN 125 Homogenizer (Cole-Parmer, Bunker Hill, IL, USA) at 9,500 rpm, twice for 15 seconds, with an interval of 30 seconds on ice. Subsequently, the homogenate was centrifuged at 20,200 g at 4°C for 30 minutes. The supernatant was collected and frozen at -80°C. Protein quantification was performed using the Bradford Protein Assay (BioRad, Hercules, CA, USA).

### Two-dimensional electrophoresis

The first dimension or isoelectric focusing (IEF) was performed in 24 cm pH 4–7 IPG strips (GE Healthcare, Little Chalfont, Buckinghamshire, UK). Initially, the strips were rehydrated for 16 hours in 450 μL rehydration solution (extraction solution containing 1,200 μg of protein, DeStreak Rehydration Solution (GE Healthcare Bio-Sciences), and 2% pH 4–7 IPG-buffer). The IEF was performed using Ettan IPGphor III System (GE Healthcare Bio-Sciences) at 20°C through the following program: step and hold until 200 V (2 h), step and hold until 500 V (1 h), gradient mode at 1,000 V (800 V/h), gradient mode at 10,000 V (16,500 V/h), and step and hold until 10,000 V (27,500 V/h). The current limit was 50 μA per strip.

For the second dimension, the strips were initially equilibrated in two successive steps of 20 minutes each, first in 5 mL of equilibration solution (6 M urea, 30% glycerol, 2% SDS, 0.002% bromophenol blue and 50 mM Tris- HCl pH 8.8) containing 1% DTT (reduction step), and then, in 5 mL of equilibration solution containing 2.5% iodoacetamide (alkylating step). Subsequently, the strips were placed on top of 1 mm thick 12.5% sodium dodecyl sulfate polyacrylamide gels and the proteins were separated using Ethan DALTsix (GE Healthcare Bio-Sciences) at 8°C. Electrophoresis was performed with 20 mA per gel for 40 minutes, followed by 40 mA per gel until the end of the run. The Low Molecular Weight Calibration Kit was used (GE Healthcare Bio-Sciences). Seven gels for each breed were made, one for each animal.

### Gel-staining and image analysis

After two-dimensional electrophoresis (2DE), the gels were immersed in fixing solution [10% acetic acid (v/v) and 50% methanol (v/v)] for 12 hours under constant shaking. Subsequently, the gels were stained with the specific fluorescent dye for phosphoproteins Pro-Q Diamond (Invitrogen Molecular Probes, Eugene, OR, USA). All procedures for phosphoproteins staining followed the optimized method described in previously suggested protocol [18]. Images of the gels with phosphoproteins were obtained using Fuji Film 5100 FLA Fluorescence Imaging System Scanner (Fuji Medical Systems, Hanover Park, IL, USA) in fluorescent scanning mode, resolution of 300 dpi, excitation filter of 532 nm and emission filter of 580 nm.

Immediately after obtaining the images of phosphorylated proteins, the gels were stained for total protein with a solution containing 8% ammonium sulfate (w/v), 0.8% phosphoric acid (v/v), 0.08% coomassie blue G-250 (v/v) and 20% methanol (v/v) [19] for 72 h and then washed with 1% acetic acid (v/v) until complete removal of excess dye. Gels were scanned using ImageScanner III (GE Healthcare Bio-Sciences) at 300 dpi and subsequently stored in 2% acetic acid (v/v) at 20°C until extraction and spots digestion.

Spots detection and quantification were performed with Image Master 2D Platinum version 7.0 software (GE Healthcare Bio-Sciences). The volume of each spot (optical density x area) was normalized to the total volume of spots detected on each gel for comparison between breeds. Differences were considered significant when *P*-value was lower than 5% by ANOVA. The comparison between breeds for each spot made by Image Master was confirmed manually. Due to the high background, the phosphoprotein image of one Nellore was not used for comparisons.

### Spots digestion and protein identification

Images of the gels stained with Pro-Q Diamond and coomassie blue G-250 were overlaid using Adobe Photoshop CC 2015.0 (Adobe Systems, San José, CA, USA) to facilitate excision of differentially abundant spots in the phosphoproteomics analysis (S1 Fig). The spots were excised manually and subjected to trypsinization [20].

Peptide mass spectra (MS and MS/MS) was obtained using matrix assisted laser desorption/ionization time-of-flight mass spectrometry (MALDI-TOF/TOF). A matrix α-cyano-4-hydroxycinnamic was used. MALDI analysis was performed using Ultraflex III MALDI-TOF/TOF system (Bruker Daltonics, Bremen, Germany). The MS analyzes were performed with reflective positive peptide method, while the MS/MS analyses were performed using the LIFT positive method.

Protein identification was made using the MASCOT version 2.2 software (Matrix Science, Boston, MA, USA) at the MS/MS ion search mode, with the following parameters: tryptic specificity, one missed cleavage and a mass measurement tolerance of 0.2 Da for MS and 0.5 Da for MS/MS mode. Cysteine carbamidomethylation was used as fixed modification, while methionine oxidation was used as variable modification. The database used was the Bovidae deposited in UniProt. The proteins identified in MASCOT were validated by SCAFFOLD version 3.6.4 software (Proteome Software, Portland, OR, USA). The criteria used for the validation was of at least one peptide, with a probability score greater than or equal to 90% for both peptides and proteins.

### Protein-protein interaction network

Differentially abundant proteins and phosphoproteins between Angus and Nellore muscle were loaded together in the String 10.0 bioinformatics software (available online: http://string-db.org/) to generate protein-protein interaction networks between the proteins identified in our study and between them and other proteins not identified here. Access numbers for each protein generated by UniProt were loaded into the software, which was set to search the *Bos taurus* database. The minimum required interaction score was set to 0.900 (highest confidence) and no more than 20 interactions were allowed in the first shell.

## Results and Discussion

A total of 423 matches ID were detected in the analysis of total protein and 1,093 in the analysis of phosphorylated proteins, of which 38 and 55 differed (*P*<0.05) between breeds, respectively. Excision of differentially abundant spots was only performed for clearly visible and separable spots on gels (36 in the proteomic analysis and 23 in the phosphoproteomic study). Due to the 2DE/MS limitations for identification of low abundance proteins, it was not possible to identify all spots that were excised.

### Differentially abundant proteins

**Fig 1:**
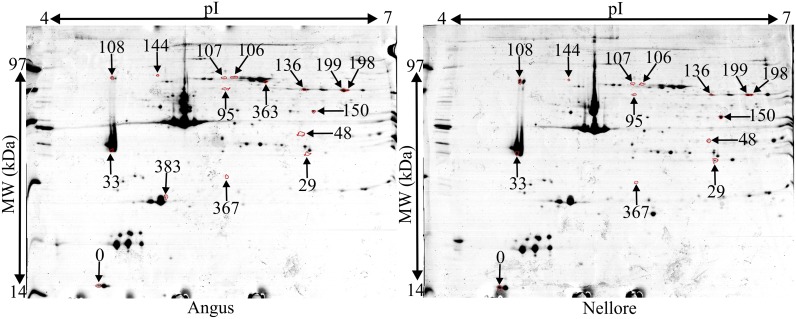
Differentially abundant proteins in the *Longissimus lumborum* muscle of Angus and Nellore bulls. Numbers found in the figure correspond to the Match ID shown in Table 1.

The proteomic analysis identified sixteen differentially abundant spots (*P*<0.05). Seven spots were more abundant in Angus and nine were more abundant in Nellore (Fig 1 and Table 1). Nellore had greater abundance of tropomyosin alpha-1 chain (TPM1, two spots), troponin T (TNNT3), myosin light chain 1 fragment (MYL1), cytoplasmic malate dehydrogenase (MDH1) and alpha-enolase (ENO1). However, Angus had greater abundance of prohibitin (PHB). Furthermore, a spot identified in Angus as myosin light chain 3 (MYL3) was not detected in Nellore (S2 Fig). Four spots were identified as phosphoglucomutase 1 (PGM1), two of them more abundant in Nellore and two in Angus. In addition, three proteins belonging to the heat shock proteins (HSPs) family were identified, two with greater abundance in Angus, mitochondrial stress-70 protein (HSPA9, two spots) and heat shock 70 kDa protein 6 (HSPA6), and one with greater abundance in Nellore, 78 kDa glucose-regulated protein (HSPA5). HSPA6 had confirmation with the realization of a blast in UniProt. Interestingly, HSPA6 was detected in only one of Nellore cattle (S3 Fig).

**Table 1:** Differentially abundant proteins in the *Longissimus lumborum* muscle of Angus and Nellore bulls.

Match ID^a^	Protein ID	ABV^b^	UniProtaccession number	Mascotscore^c^	Foldchange	*P*-value^d^	% Protein identification probability^e^	Identified peptides^f^	% Proteincoverage^g^	pI observed/expected	MW kDa observed/expected
More abundant in Angus											
383	Myosin light chain 3	MYL3	P85100	188	ND^h^ in Nellore	0.0007	100	2	13	5.12/5.00	25.7/22.1
136	Phosphoglucomutase-1	PGM1	Q08DP0	233	2.83	0.0031	100	3	8	6.30/6.36	63.1/61.8
199	Phosphoglucomutase-1	PGM1	Q08DP0	933	2	0.0079	100	11	24	6.64/6.36	62.6/61.8
106	Stress-70 protein, mitochondrial	HSPA9	Q3ZCH0	86	1.64	0.0111	99	1	2	5.72/5.97	75.8/74.0
107	Stress-70 protein, mitochondrial	HSPA9	Q3ZCH0	264	2.03	0.0183	100	3	6	5.63/5.97	75.9/74.0
363	Uncharacterized protein	HSPA6	F1MWU9	90	4.6	0.0349	99	1	2	5.98/5.74	68.3/71.4
367	Prohibitin	PHB	Q3T165	255	2.15	0.0238	100	3	13	5.65/5.57	29.7/29.8
More abundant in Nellore											
0	Myosin light chain 1 (fragment)	MYL1	Q08E10	209	1.89	0.0140	100	3	18	4.50/4.73	14.4/19.7
108	Tropomyosin alpha-1 chain	TPM1	Q5KR49	635	2.93	0.0197	100	7	25	4.66/4.69	75.3/32.7
33	Tropomyosin alpha-1 chain	TPM1	Q5KR49	337	1.72	0.0422	100	5	15	4.63/4.69	35.9/32.7
48	Troponin T	TNNT3	Q8MKI3	91	1.96	0.0084	99	1	5	6.30/5.99	40.3/32.1
29	Malate dehydrogenase, cytoplasmic	MDH1	Q3T145	57	2.06	0.0009	95	1	4	6.34/6.16	35.2/36.7
150	Alpha-enolase	ENO1	Q9XSJ4	861	2.04	0.0152	100	11	29	6.39/6.37	49.0/47.6
198	Phosphoglucomutase-1	PGM1	Q08DP0	711	3.28	0.0079	100	8	19	6.67/6.36	63.4/61.8
95	Phosphoglucomutase-1	PGM1	A6QPB5	157	1.92	0.0160	100	1	3	5.64/5.48	65.2/62.5
144	78 kDa glucose-regulated protein	HSPA5	Q0VCX2	303	2	0.0131	100	4	7	5.07/5.07	78.7/72.4

^a^ Match ID correspond to the numbers shown in Fig 1

^b^ Protein abbreviation is in accordance with gene abbreviation in UniProt

^c^ The Mascot baseline significant score is 31

^d^ *P*-value obtained by ANOVA comparing spots abundance between Angus and Nellore muscle

^e^ Probability for validation by Scaffold software of the proteins identified by Mascot

^f^ Number of peptides identified in Mascot and validated by Scaffold. The sequences of the peptides are found in S1 Table

^g^ Protein coverage calculated by Scaffold (identified amino acids / total amino acids)

^h^ ND spot not detected

### Differentially abundant phosphoproteins

**Fig 2:**
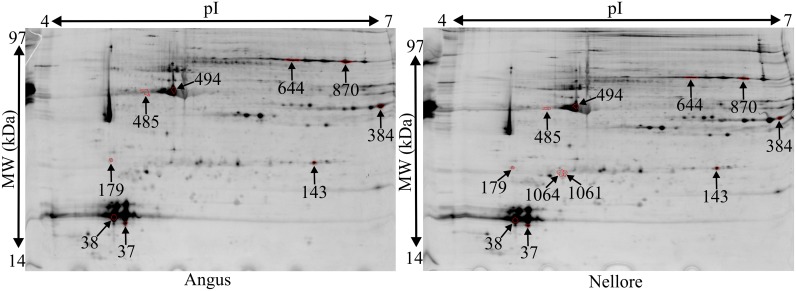
Differentially abundant phosphoproteins in the *Longissimus lumborum* muscle of Angus and Nellore bulls. Numbers found in the figure correspond to the Match ID shown in Table 2.

The phosphoproteomic approach identified eleven differentially phosphorylated spots (*P*<0.05), three more abundant in Angus and eight more abundant in Nellore (Fig 2 and Table 2). There were two spots of myosin light chain 1/3 (MYL1) that were detected only in Nellore (S4 Fig). Additionally, Nellore had higher phosphorylation of myosin regulatory light chain 2 (MYLPF, two spots), alpha actin 1 (ACTA1, two spots), triosephosphate isomerase (TPI1) and 14-3-3 protein epsilon (YWHAE). However, Angus had greater phosphorylation of PGM 1 (two spots) and TNNT3.

**Table 2:** Differentially abundant phosphoproteins in the *Longissimus lumborum* muscle of Angus and Nellore bulls.

Match ID^a^	Protein ID	ABV^b^	Uniprotaccession number	Mascotscore^c^	Foldchange	*P*-value^d^	% Protein identification probability^e^	Identified peptides^f^	% Proteincoverage^g^	pI observed/expected	MW kDa observed/expected
More abundant in Angus											
384	Troponin T	TNNT3	Q8MKI4	103	3.56	0.0411	100	2	10	6.91/5.93	39.8/32.1
644	Phosphoglucomutase-1	PGM1	Q08DP0	199	1.65	0.0065	100	3	8	6.19/6.36	64.9/61.6
870	Phosphoglucomutase-1	PGM1	Q08DP0	377	1.95	0.0481	100	6	10	6.63/6.36	63.6/61.8
More abundant in Nellore											
1061	Myosin light chain 1/3	MYL1	A0JNJ5	412	ND^h^ in Angus	0.0001	100	5	34	5.12/4.96	24.8/21.0
1064	Myosin light chain 1/3	MYL1	A0JNJ5	80	ND^h^ in Angus	0.0001	99	1	5	5.07/4.96	25.0/21.0
38	Myosin regulatory light chain 2	MYLPF	Q0P571	212	1.45	0.0063	100	3	24	4.69/4.91	18.1/19.0
37	Myosin regulatory light chain 2	MYLPF	Q0P571	113	1.26	0.0213	100	1	7	4.79/4.91	17.6/19.0
485	Alpha actin 1	ACTA1	A4IFM8	465	1.82	0.0290	100	6	22	4.96/5.23	43.2/42.3
494	Alpha actin 1	ACTA1	A4IFM8	213	1.29	0.0470	100	2	6	5.22/5.23	43.9/42.3
143	Triosephosphate isomerase	TPI1	Q5E956	550	1.31	0.0113	100	6	34	6.40/6.45	26.4/26.9
179	14-3-3 protein épsilon	YWHAE	P62261	219	1.25	0.0228	100	3	12	4.67/4.63	26.8/29.3

^a^ Match ID correspond to the numbers shown in Fig 2

^b^ Protein abbreviation is in accordance with gene abbreviation in UniProt

^c^ The Mascot baseline significant score is 31

^d^ *P*-value obtained by ANOVA comparing spots abundance between Angus and Nellore muscle

^e^ Probability for validation by Scaffold software of the proteins identified by Mascot

^f^ Number of peptides identified by Mascot and validated by Scaffold. The sequences of the peptides are found in S2 Table

^g^ Protein coverage calculated by Scaffold (identified amino acids / total amino acids)

^h^ ND spot not detected

Only one differentially phosphorylated spot was also differentially abundant in proteomics analysis. It was the spot 870 (PGM1), which was more abundant in Angus and corresponded to spots 198 and 199 (PGM1) in proteomics analysis, which were more abundant in Nellore and Angus, respectively. The other differentially abundant spots in phosphoproteomics study showed no significant difference in proteomic analysis among Angus and Nellore. Thus, the differences in phosphoproteins observed could be attributed to differences in phosphorylation level instead of amount in total protein.

### Biological processes related to identified proteins and phosphoproteins and protein-protein interaction network

**Fig 3:**
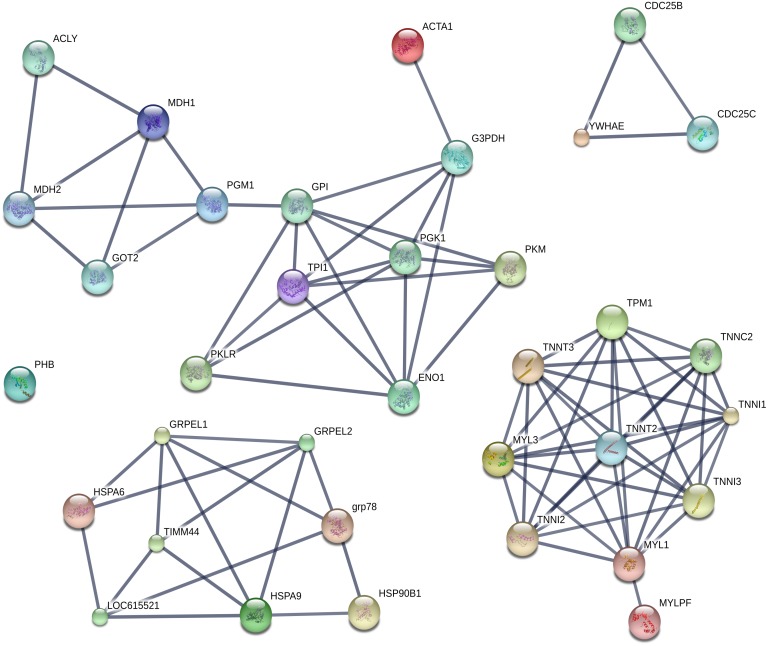
Protein—protein interaction analysis of differentially abundant proteins and phosphoproteins between Angus and Nellore muscle. Data have been elaborated and graphed through String 10.0. ATP-citrate synthase (ACLY), Mitochondrial malate dehydrogenase (MDH2), Cytoplasmic malate dehydrogenase (MDH1), Mitochondrial aspartate aminotransferase (GOT2), Phosphoglucomutase-1 (PGM1), Glucose-6-phosphate isomerase (GPI), Triosephosphate isomerase (TPI1), Pyruvate kinase (PKLR), Alpha-enolase (ENO1), M1-type pyruvate kinase (PKM), Phosphoglycerate kinase 1 (PGK1), Glyceraldehyde-3-phosphate dehydrogenase (G3PDH), Alpha skeletal muscle actin (ACTA1), Uncharacterized protein (CDC25B), M-phase inducer phosphatase 3 (CDC25C), 14-3-3 protein epsilon (YWHAE), Myosin regulatory light chain 2 (MYLPF), Myosin light chain 1/3 (MYL1), Cardiac troponin I (TNNI3), Uncharacterized protein (TNNI1), Fast troponin C type 2 (TNNC2), Tropomyosin alpha-1 chain (TPM1), Troponin T fast skeletal muscle type (TNNT3), Myosin light chain 3 (MYL3), TNNI2 protein (TNNI2), Cardiac troponin T (TNNT2), Mitochondrial GrpE protein homolog 1 (GRPEL1), Mitochondrial GrpE protein homolog 2 (GRPEL2), 78 kDa glucose-regulated protein (GRP78), Endoplasmin (HSP90B1), Mitochondrial stress-70 protein (HSPA9), Mitochondrial import inner membrane translocase subunit TIM44 (TIMM44), LOC615521 protein (LOC615521), Uncharacterized protein (HSPA6).

The main biological processes related to proteins and phosphoproteins identified in this study are summarized in Table 3. In addition, the protein-protein interaction map obtained through differentially abundant proteins and phosphoproteins between Angus and Nellore muscle using String 10.0 is shown in Fig 3. Proteins and phosphoproteins were loaded together in the analysis to obtain a more robust interaction network. Highly reliable relationships between the proteins identified in this study and among them and other unidentified proteins were obtained using this bioinformatics tool.

**Table 3:** Biological processes related to differentially abundant proteins and phosphoproteins in the *Longissimus lumborum* muscle of Angus and Nellore bulls.

Protein ID	ABV^a^	Biological process described in UniProt^b^
14-3-3 protein épsilon	YWHAE	Negative regulation of peptidyl-serine dephosphorylation; Protein targeting; Regulation of membrane repolarization; Regulation of potassium ion transmembrane transporter activity
Prohibitin	PHB	DNA biosynthetic process; Mitochondrion organization; Negative regulation of protein catabolic process; Negative regulation of transcription by competitive promoter binding; Protein stabilization
Stress-70 protein, mitochondrial	HSPA9	Protein export from nucleus; Protein folding
Uncharacterized protein	HSPA6	Cellular response to heat; Protein refolding
78 kDa glucose-regulated protein	HSPA5	Maintenance of protein localization in endoplasmic reticulum
Malate dehydrogenase, cytoplasmic	MDH1	Carbohydrate metabolic process; Malate metabolic process; NADH metabolic process; Oxaloacetate metabolic process; Tricarboxylic acid cycle
Alpha-enolase	ENO1	Glycolytic process
Phosphoglucomutase-1	PGM1	Glucose metabolic process; Glycogen biosynthetic process
Triosephosphate isomerase	TPI1	Gluconeogenesis; Glyceraldehyde-3-phosphate biosynthetic process; Glycerol catabolic process; Glycolytic process; Pentose-phosphate shunt
Myosin light chain 3	MYL3	Regulation of striated muscle contraction
Myosin light chain 1/3	MYL1	Muscle contraction
Myosin regulatory light chain 2	MYLPF	Skeletal muscle tissue development
Alpha actin 1	ACTA1	Positive regulation of gene expression; Skeletal muscle fiber development; Skeletal muscle thin filament assembly
Tropomyosin alpha-1 chain	TPM1	Regulation of striated muscle contraction;
Troponin T	TNNT3	Regulation of ATPase activity; Regulation of striated muscle contraction; Skeletal muscle contraction

^a^Protein abbreviation is in accordance with gene abbreviation in UniProt

^b^Information obtained through UniProt accession numbers shown in Tables 1 and 2

Three major clusters were distinguished: Energy metabolism-related proteins (ACLY, MDH2, MDH1, GOT2, PGM1, GPI, TPI1, PKLR, ENO1, PKM, PGK1, G3PDH and ACTA1); proteins involved in the regulation of muscle contraction (MYLPF, MYL1, TNNI3, TNNI1, TNNC2, TPM1, TNNT3, MYL3, TNNI2 and TNNT2) and proteins related to protein folding, protein import into mitochondrial matrix and cellular stress response (GRPEL1, GRPEL2, GRP78, HSP90B1, HSPA9, TIMM44, LOC615521 and HSPA6). A small interaction network involving the phosphatases CDC25B and CDC25C and the adapter protein YWHAE was also obtained. PHB was the only protein identified in this study that did not show significant interactions. This result could be related to lack of information on PHB interactions in *Bos taurus* database and/or strict settings used in our analysis.

### Differences related to meat quality

The Nellore and Angus cattle used in our study showed significant differences in the main characteristics related to meat quality, which were evaluated in parallel studies of our research group. Nellore had tougher beef as measured by Warner—Bratzler shear force (7.6 ± 0.33 vs. 6.9 ± 0.33 kgf) and lower myofibrillar fragmentation indices (MFI, 47.6 ± 4.28 vs. 69.2 ± 4.28%) [21]. Furthermore, Nellore had lower content of intramuscular fat (IMF, 2.7 ± 0.08 vs. 3.4 ± 0.08% ether extract). However, the content of total intramuscular collagen did not differ between Angus and Nellore [6].

### Nellore had greater abundance of proteins involved in the regulation of muscle contraction, which are important substrates of proteolytic enzymes during the meat aging

TNNT3 [15] and TPM1 [22] have been more abundant in tough beef. These findings are in agreement with our results, because Nellore muscle showed greater abundance of TPM1 and TNNT3 compared to Angus. Tropomyosin (Tpm) and troponin T (TnT) are among the main substrates of the proteolytic enzymes related to meat tenderization [23], and TnT degradation during aging has been positively associated with beef tenderization [17, 24].

In addition, due to the role of TnT in the regulation of the complex that controls the interaction among actin and myosin filaments, it has been suggested that changes in the relation between them could occur in response to TnT degradation [25]. The degradation of TnT could also be related to the disruption of its interaction with other thin filaments and, consequently, with the breaking of thin filaments in the sarcomeric I-band, which might lead to fragmentation of myofibrils. This suggests that differences in the beef tenderness between Angus and Nellore could be partly related to differences in the muscle abundance of TNNT3 and TPM1, which are involved in regulating contraction and muscle structure organization [11].

Interestingly, the spot 108 that was identified as TPM1 had a molecular weight that was approximately twice the theoretical value (75.3 versus 32.7 kDa). Because TPM1 is a dimer of α-helices forming a coiled-coil [26], this result might indicate the presence of TPM1 dimer despite the denaturing conditions of electrophoresis. The presence of dimers in 2DE analysis has already been suggested [12].

### Myofilaments expressed in fast or slow twitch fibers had different abundance between Angus and Nellore

MYL1 and MYL3 are the regulatory light chain of myosin. Nellore had increased abundance of MYL1, which is found in fast twitch fibers, while MYL3, which is found in slow twitch fibers, was only detected in Angus. Although we have not evaluated the proportion of muscle fiber types between breeds, the differences in the abundance of myosins expressed in fast or slow twitch fibers could suggest that muscle fiber type might have differed between Angus and Nellore. Likewise, TNNT3, an isoform also expressed in fast twitch fibers, was most abundant in Nellore. This is in line with a prior study, that observed lower proportion of fast twitch oxidative glycolytic fibers and higher of slow twitch oxidative fibers in the *Longissimus dorsi* muscle of crossbred Angus×Nellore compared to Nellore cattle [27]. In addition, Angus is considered to have a higher proportion of oxidative muscle fibers [28]. Muscle fiber type has been associated with meat quality [29]. However, more studies are needed to assess whether muscle fiber type is related to differences in beef quality between Angus and Nellore.

Similar to what was observed in our study, crossbred Angus×Holstein Friesian had lower abundance of MYL1 in *Longissimus lumborum* muscle than crossbred Belgian Blue×Holstein Friesian cattle, which were characterized by later body maturity and leaner carcasses [16]. In addition, Large White pigs (leaner carcass) had greater MYL1 abundance than Casertana pigs (fatter carcass) [30], and pigs with higher IMF content had lower abundance of fast twitch myofilaments and greater abundance of slow twitch myofilaments [31]. Furthermore, our results were also consistent with those obtained in a similar study, in which Podolian cattle had higher abundance of TnT and MYL1, tougher beef and lower MFI and IMF content than crossbreed Romagnola×Podolian and Friesian cattle [17]. Likewise, MYL1 was more abundant in Chianina cattle classified as tough beef [13].

### Differences in abundance of enzymes involved in muscle energy metabolism between Angus and Nellore

Nellore had greater ENO1 abundance, a glycolytic enzyme that catalyzes the conversion of 2-phosphoglycerate to phosphoenolpyruvate. This result is in agreement with the higher abundance in Nellore of myofilaments expressed in fast twitch glycolytic fibers. Similarly, ENO1 was more abundant in steers compared to bulls and in *Semitendinosus* compared to *Longissimus thoracis* muscle, and this was consistent with the greater proportion of fast twitch glycolytic fibers reported for steers and *Semitendinosus* muscle [32].

Two spots identified as PGM1 were more abundant in Nellore, while two other spots, also identified as PGM1 were more abundant in Angus. PGM1 is involved in glycolysis and glycogenesis reversibly catalyzing the conversion of glucose 1-phosphate to glucose 6-phosphate. This could be related to metabolic differences in muscle tissue between breeds before slaughter. Glycogen synthesis and glucose degradation are both intense metabolic activities expected in muscles with a greater amount of fast twitch myofilaments [33], as it was observed in Nellore. Likewise, as the catalysis of glucose is the major metabolic pathway for the supply of carbon and reduced cofactor to the synthesis of intramuscular fat [16, 30, 34], it was also expected great abundance of PGM1 in Angus, because they had higher IMF content. Similarly, cattle with greater genomic estimated breeding value for IMF had higher expression of PGM1 [35].

Furthermore, Nellore had higher abundance of MDH1, an enzyme important in gluconeogenesis that catalyzes the oxidation of malate to oxaloacetate, which may then be used as precursor for glucose synthesis. This result is in line with what has already been discussed for ENO1 and PGM1.

The activity of enzymes involved in energy metabolism is of great importance for the meat quality characteristics, because the formation of actin-myosin complex and pH drop are the main changes responsible for the conversion of muscle to meat, and they are strongly influenced by postmortem energy metabolism [10, 25]. However, the relation between abundance of energy metabolism enzymes and meat tenderness has been a controversial topic [10, 15, 32]. This discrepancy could be related to differences in enzyme activity, that may be altered by post-translational modifications such as phosphorylation [7, 12, 13].

### Angus and Nellore differed in the abundance of heat shock proteins located in the mitochondria or sarcoplasmic reticulum that are involved in Ca^2+^cellular traffic and apoptosis

Several studies have found a relationship between meat tenderness and HSPs abundance [22, 36, 37]. Due to the highly conserved chaperone capacity of proteins belonging to the HSPs family, many research groups have discussed the involvement of HSPs in the meat tenderizing process, without considering their singularities. In our study, four spots belonging to the HSPs family were identified, three more abundant in Angus and one in Nellore. Because HSPs have different cell locations, respond to different stimuli, and have different activities, we will discuss them separately to give greater biological significance to our results.

HSPA9 also known as mortalin and 75 kDa glucose-regulated protein (GRP-75) is the main mitochondrial HSP. It plays a key role in the translocation system that imports and exports protein across the mitochondrial membrane [38]. Despite belonging to the HSPs family, the expression of HSPA9 does not increase in response to heat stress. However, it is stimulated by glucose deprivation, Ca^2+^ influx and some cytotoxins [39]. Although HSPA9 is associated with anti-apoptotic processes due to its chaperone activity and inhibition of pro-apoptotic factors, it has also been proposed that under conditions of excessive stress, HSPA9 could not be able to prevent cell death and would change the mitochondrial functions leading to apoptosis [39].

HSPA9 abundance in *Longissimus thoraci* [40] and *Semitendinosus* [41] muscle immediately after slaughter had positive correlation with beef tenderness. These findings were in agreement with our result, as Angus had higher beef tenderness and greater HSPA9 abundance. This effect of HSPA9 on meat tenderness might be related to its anti-apoptotic effect, preventing the formation of protein aggregates, and might also be related to its role in triggering apoptosis. After slaughter and exsanguinations, muscle cells are subjected to various conditions of acute stress, such as interruption of the blood supply of glucose and oxygen, pH drop due to lactic acid accumulation, and increased Ca^2+^concentration in sarcoplasm and mitochondria [10]. All these conditions may induce apoptosis, which has been described as one of the main mechanism responsible for the meat tenderization through caspase proteolytic system [42].

Several mechanisms may involve HSPA9 in the triggering of apoptosis. For example, HSPA9 connects the inositol 1,4,5-trisphosphate receptor of the sarcoplasmic reticulum to the voltage-dependent anion channels of the mitochondria, facilitating the transfer of Ca^2+^ from the sarcoplasmic reticulum into the mitochondria. Overloading of Ca^2+^ in the mitochondria could lead to depolarization of the inner mitochondrial permeability transition pore and trigger apoptosis [43]. Additionally, the release of Ca^2+^ into the sarcoplasm leads to mitochondrial uptake of Ca^2+^ that cause conformational changes in the outer membrane-bound mitochondrial m-calpain large subunit, which leads to its binding to calpain small subunit and HSPA9. The formation of this complex allows the translocation of mitochondrial m-calpain from the outer membrane into the intermembrane space, where it is activated by a further increase of mitochondrial Ca^2+^ level triggering apoptosis [44].

Unlike what was observed for HSPA9, Nellore had greater abundance of HSPA5. This is the main HSP located in the sarcoplasmic reticulum, where is essential for the transport of newly synthesized polypeptides, for the folding and assembly of proteins, and for Ca^2+^ homeostasis [45]. HSPA5 has chaperone activity stimulated by its binding to Ca^2+^ and participates in the Ca^2+^ buffering in the sarcoplasmic reticulum. Ca^2+^ connected to HSPA5 corresponds to 25% of the Ca^2+^ reserves in the sarcoplasmic reticulum [46]. When Ca^2+^ reserves decrease or are depleted, there is a greater amount of unfolded proteins in the sarcoplasmic reticulum, which leads to increased expression of HSPA5 [47]. HSPA5 has been mainly related to inhibition of sarcoplasmic reticulum stress-related apoptosis [45]. To the best of our knowledge, HSPA5 has not been previously associated with meat tenderness differences.

The divergence in the abundance of HSPA9 and HSPA5 between Angus and Nellore was intriguing, as both proteins are related to cell flow of Ca^2+^. After slaughter, Ca^2+^ retained in the sarcoplasmic reticulum is released into the sarcoplasm stimulating the rigor mortis and the calpain activity, which is considered one of the main responsible for myofibrillar degradation and meat tenderization during aging [23, 25]. In addition, the output of Ca^2+^ from the sarcoplasmic reticulum to other cell compartments such as mitochondria triggers apoptosis [48].

A model has been proposed to explain how the Ca^2+^ flow could integrate the sarcoplasmic reticulum with the mitochondrial function [43]. In this model, HSPA5 is involved keeping Ca^2+^ within the sarcoplasmic reticulum, while HSPA9 is involved in the communication of sarcoplasmic reticulum and mitochondria that directs the Ca^2+^ flow from the former to the latter. Furthermore, it was suggested that the balance between the Ca^2+^ amount in the mitochondria and sarcoplasmic reticulum would be determinant to the decision between cell survival or death, wherein the Ca^2+^ overload within the mitochondria would direct to apoptosis. The massive Ca^2+^ influx into the matrix leads to mitochondria fission and accelerates the release of cytochrome c amplifying apoptosis via activation of caspases [8].

In this way, we could hypothesize that the greater abundance of HSPA5 in Nellore would delay, while the higher abundance of HSPA9 in Angus would accelerate apoptosis, rigor mortis, and beef tenderization. In agreement with this, HSPA9 level in muscle after slaughter had negative correlation with both pH at 3 hours and ultimate pH in the cattle carcass, and it was proposed that this result would be associated with an increased release of Ca^2+^ from the sarcoplasmic reticulum and, consequently, with higher enzyme activity and rigor mortis [49]. A positive relation found between μ-calpain and HSPA9 through correlation networks among protein biomarkers of beef tenderness also support our hypothesis [50]. Furthermore, it has been suggested that calcium-binding proteins, such as HSPA5, could contribute to the lower amount of free calcium after slaughter and, consequently, in reduced calpain activity [10].

Intriguingly, the calpastatin gene knockdown in bovine muscle satellite cells significantly increased the mRNA expression of μ-calpain, caspases and heat shock proteins, suggesting that they are involved in apoptosis during the calpastatin gene silencing [51]. Additionally, it has been reported that caspase-3, an effector enzyme of apoptosis, could inhibit the calpastatin activity, which is the calpain inhibitor [11]. The high calpastatin activity has been considered one of the main factors related to lower zebu beef tenderness compared to taurine [52]. Differences in the abundance of calpastatin, calpain and caspases were not detected in our study. This result may be attributed to limitations of 2DE to detect differences in low-abundance proteins [12]. However, in a parallel study with the same animals used here, there was a higher calpastatin activity in Nellore beef [21]. In view of this, we could suggest that the calpain/calpastatin proteolytic system and caspase-dependent apoptosis together would be related to differences in beef tenderness between Angus and Nellore. Furthermore, a greater susceptibility to caspase-dependent apoptosis would be related to lower calpastatin activity and greater MFI in Angus. However, more investigations are needed to evaluate this hypothesis.

As HSPA5 and HSPA9 are mainly located within the sarcoplasmic reticulum and mitochondria, respectively, the higher abundance of HSPA5 in Nellore and the greater abundance of HSPA9 in Angus would also be related to differences in muscle fiber types among them, since fast twitch glycolytic fibers have higher volume of sarcoplasmic reticulum, while slow twitch oxidative fibers have greater mitochondrial volume and abundance [53].

Another HSP that differed between Angus and Nellore was HSPA6, which was more abundant in Angus and was detected only in one of Nellore cattle. HSPA6 expression has been reported to be strictly stimulated by heat in fibroblasts [54]. In another study, HSPA6 expression was strongly induced by heat, but it had no significant effect on protection of HEK-293 cells against heat-induced cell death [55]. These findings are interesting because in a parallel study evaluating the same animals used in the current study, there was higher metabolic heat production and higher body temperatures in Angus [56].

### Prohibitin seems to be a potential biomarker of intramuscular fat content in cattle

PHB are part of a group of proteins highly conserved and ubiquitously expressed in different cell tissues, being mainly located in the mitochondria, nucleus and plasma membrane [57]. Due to its location in several cellular compartments, translocation and interaction capacity with many transcription factors and proteins, PHB is involved in regulation of cell survival, apoptosis, metabolism and inflammation [58]. It may be upregulated under conditions of extreme stress and lead to apoptosis by modulating transcription factors and pro-apoptotic genes increasing caspases activity [59, 60].

In our study, PHB was more abundant in Angus. A greater abundance of PHB has already been described in bovine muscle classified as tender beef [61]. Furthermore, it was also observed by these authors higher abundance of other proteins of the inner and outer mitochondrial membranes, such as HSPA9, in the muscle of tender beef, which would be related to apoptosis. These findings support our suggestion that the difference in beef tenderness between Angus and Nellore would be partially explained by differences in apoptosis.

In addition to its relation with differences in meat tenderness, PHB could also be involved with differences in the IMF content between Angus and Nellore. It has been proposed that PHB would regulate adipocyte differentiation by modulating the insulin signaling pathway and mitochondrial biogenesis. Moreover, PHB also would regulate lipogenesis by modulating the pyruvate carboxylase and mitochondrial function [62]. PHB upregulation resulted in adipocyte hypertrophy associated with increase of white adipose tissue in mice [63]. Treatment of fibroblasts with insulin or peroxisome proliferator-activated receptor gamma (PPAR-γ) caused PHB upregulation and induced adipogenesis with increased expression of PPAR-γ [62]. These findings are interesting, because in a parallel study, there was greater abundance of PPAR-γ in Angus muscle [6]. In addition, higher PHB abundance in IMF compared to subcutaneous, perirenal, and intermuscular fat has been observed in pigs [64]. These findings together with our results suggest that differences in PHB abundance might partially explain the lower deposition of IMF in Nellore compared to Angus. Additionally, PHB could be considered as a potential biomarker of IMF in cattle.

### Angus and Nellore differed in phosphorylation of myofilaments, which is related to affect muscle contraction strength and susceptibility to calpain and apoptosis

Nellore presented higher phosphorylation of MYLPF, MYL1 and ACTA1, while Angus had only increased TNNT3 phosphorylation. MYLPF phosphorylation might alter the structure and motor function of the myosin to increase the sensitivity of the contractile apparatus to Ca^2+^ [65]. Furthermore, MYLPF phosphorylation increased the contraction force in fast twitch skeletal muscle [66]. In addition, it has been suggested that phosphorylation of MYLPF might work as a kind of memory to enhance muscle contraction strength [67]. This hypothesis has been considered in an attempt to explain the relationship between tough meat and MYLPF phosphorylation [11]. These findings and hypotheses are consistent with our results, as Nellore had greater phosphorylation of MYLPF and tougher beef. A similar result was observed in a study with sheep, in which there was higher MYLPF phosphorylation in the group of animals classified as tough meat [14]. Moreover, greater phosphorylated MYLPF abundance has been reported in dark firm dry beef [68].

The phosphorylated MYLPF is expressed in fast twitch fibers. Since there was no difference in abundance of this protein in proteomic analysis, we suggest that the difference found would be related to differences in phosphorylation and or MYLPF dephosphorylation more than a possible difference in the fast and slow twitch fibers composition between Angus and Nellore. MYLPF is phosphorylated by Ca^2+^/calmodulin-dependent myosin light chain kinase and is dephosphorylated by protein phosphatase 1 [69]. Despite the involvement of Ca^2+^ in MYLPF phosphorylation mechanism, this process does not require high Ca^2+^ concentrations to occur. Other factors such as myosin light chain kinase:protein phosphatase-1 ratio appear to be important to affect the MYLPF phosphorylation [67]. Additionally, it was demonstrated that myosin light chain kinase rather than calmodulin is limiting to the phosphorylation of MYLPF [66].

Although Angus had lower TNNT3 abundance in proteomic study, they showed greater phosphorylation of another TNNT3 isoform. Phosphorylation of skeletal troponins increased their susceptibility to degradation by calpain possibly due to dissociation from the native complex [70]. In addition, it has been suggested that TnT would undergo cut-off in phosphorylated sites during post-rigor stage [71]. TnT is one of the main substrates for calpain and its degradation is related to the meat tenderization during aging [23]. Therefore, we could suggest that the greater phosphorylation of TNNT3 in Angus would partly explain its higher MFI and beef more tender compared to Nellore. Additionally, the lower TNNT3 phosphorylation in Nellore would be partially explained by greater TPM1 abundance observed in these animals, as was shown in proteomics analysis, because skeletal Tpm may inhibit the phosphorylation of skeletal TnT due to the strong interaction between them, reducing the exposure of TnT phosphorylation sites [72].

ACTA1 was other myofilament that had different phosphorylation level between Angus and Nellore. Likewise to what was observed in our study, higher level of ACTA1 phosphorylation was found in tough beef [13]. It has been suggested that phosphorylation of ACTA1 could prevent the onset of apoptosis and would be positively correlated with the meat toughness [11]. Our result and these findings give further support to our hypothesis that apoptosis would be involved in the difference of beef tenderness between Angus and Nellore.

### Two enzymes involved in glucose metabolism had opposite levels of phosphorylation in Angus and Nellore

Angus and Nellore had greater abundance of PGM1 isoforms in the proteomics analysis, but the phosphoproteomics revealed higher PGM1 phosphorylation only in Angus. PGM1 is more active when phosphorylated due to a conformational change that exposes its active site in response to phosphorylation [73]. As previously discussed, this protein catalyzes reactions that drive glucose into glycolysis or glycogenesis. As muscle does not receive more nutrients after slaughter and as glycolysis becomes the major source of energy to the muscle cells, we could suggest that greater PGM1 phosphorylation would contribute to a faster glycolysis in Angus. Supporting this hypothesis, it was proposed that phosphorylation of PGM1 is related to faster rates of glycolysis and pH drop in postmortem muscle [74]. Furthermore, an increase in the phosphorylated PGM1 abundance was observed in cattle muscle from 0 to 1 day after slaughter, which would be related to an increase in glycogenolysis and glycolysis due to increased anaerobic postmortem muscle metabolism [75].

On the other hand, TPI1 phosphorylation was higher in Nellore. Similarly to our study, TPI1 was more phosphorylated in tough beef [12]. TPI1 is a glycolytic enzyme that catalyzes the reversible conversion of D-glyceraldehyde 3-phosphate from glycerone phosphate. The phosphorylation of TPI1 decreased its activity in HeLa cells [76]. In addition, it was reported a higher abundance of phosphorylated TPI1 in the pigs muscle with slow pH decline compared to fast pH decline group [77]. A moderate rate of pH decline in cattle muscle could be beneficial to meat tenderness due to the lower risk of cold shortening and influence on the activity of proteolytic enzymes [25].

### YWHAE phosphorylation might also be involved with differences in force of muscle contraction and apoptosis between Angus and Nellore

YWHAE belongs to 14-3-3 protein family working as adapters in the regulation of several signaling pathways due to their abilities to bind to a large number of proteins. It has been suggested that phosphorylation of 14-3-3 proteins might result in dimer formation or dissociation, and it might also cause changes in their binding sites, which would modulate their interaction with target proteins [78]. Specifically, we did not find studies about the effect of phosphorylation on the regulation of YWHAE. Anyway, it has been well documented that YWHAE negatively regulates apoptosis [79]. In addition, it was proposed that 14-3-3 proteins might bind to phosphorylated myosin light chain kinase and this could influence its binding to myosin [80].

Because Nellore had greater phosphorylation of YWHAE and tougher beef, we could hypothesize that YWHAE phosphorylation would affect beef tenderness preventing apoptosis and enhancing the strength of muscle contraction. Additionally, this result might be involved with the difference in phosphorylation of MYLPF through its probable effect on the myosin light chain kinase activity. Other studies had already suggested the involvement of 14-3-3 proteins with the meat tenderness due to their likely involvement in apoptosis and muscle contraction force [15, 30].

## Conclusions

We can conclude that differences in proteins involved with contraction and muscle organization, myofilaments expressed in fast or slow-twitch fibers and heat shock proteins localized in mitochondria or sarcoplasmic reticulum and involved in cell flux of calcium and apoptosis might be associated with differences in beef quality between Angus and Nellore. Furthermore, prohibitin appears to be a potential biomarker of intramuscular fat content in cattle. In addition, differences in phosphorylation of myofilaments and glycolytic enzymes could be involved in differences in muscle contraction force, susceptibility to calpain, apoptosis and postmortem glycolysis, which might also be related to differences in beef quality among Angus and Nellore.

This was the first proteomic and phosphoproteomic approach comparing taurine and zebu muscle and, among the new findings, we could highlight the possible importance of apoptosis for differences in beef tenderness between Angus and Nellore. Thus, we could suggest further studies to evaluate if a possible difference in apoptosis susceptibility among taurine and zebu muscle would be related to the difference in the calpain/calpastatin system, which is currently considered the main cause of difference in beef tenderness between them.

## Supporting Information

S1 Fig Overlap of images of a gel stained with Pro-Q Diamond and Coomassie blue G-250. The image obtained with Pro-Q Diamond was colored in blue and the image obtained with Coomassie blue G-250 was colored in red. The overlap of spots in the two images produced brown color.
(TIF)

S2 Fig Spot identified as myosin light chain 3 (MYL3). Spots highlighted in the green square (match ID 383) and only detected in Angus muscle.
(TIF)

S3 Fig Spot identified as heat shock 70 kDa protein 6 (HSPA6). Spots highlighted in the green square (match ID 363) and only detected in the muscle of one of Nellore cattle.
(TIF)

S4 Fig Two spots identified as myosin light chain 1/3 (MYL1). Spots highlighted in the green square (match IDs 1064 and 1061) and only detected in Nellore muscle.
(TIF)

S1 Table Differentially abundant proteins between Angus and Nellore cattle muscle. Sequence of the peptides identified in Mascot and validated by the Scaffold.
(DOCX)

S2 Table Differentially abundant phosphoproteins between Angus and Nellore cattle muscle. Sequence of the peptides identified in Mascot and validated by the Scaffold.
(DOCX)
