# Supplementary figures and images for: Differences in Beef Quality between Angus (Bos taurus taurus) and Nellore (Bos taurus indicus) Cattle through a Proteomic and Phosphoproteomic Approach
Source: PLoS One. 2017 Jan 19;12(1):e0170294. doi: 10.1371/journal.pone.0170294 (PMC5245812; doi:10.1371/journal.pone.0170294)

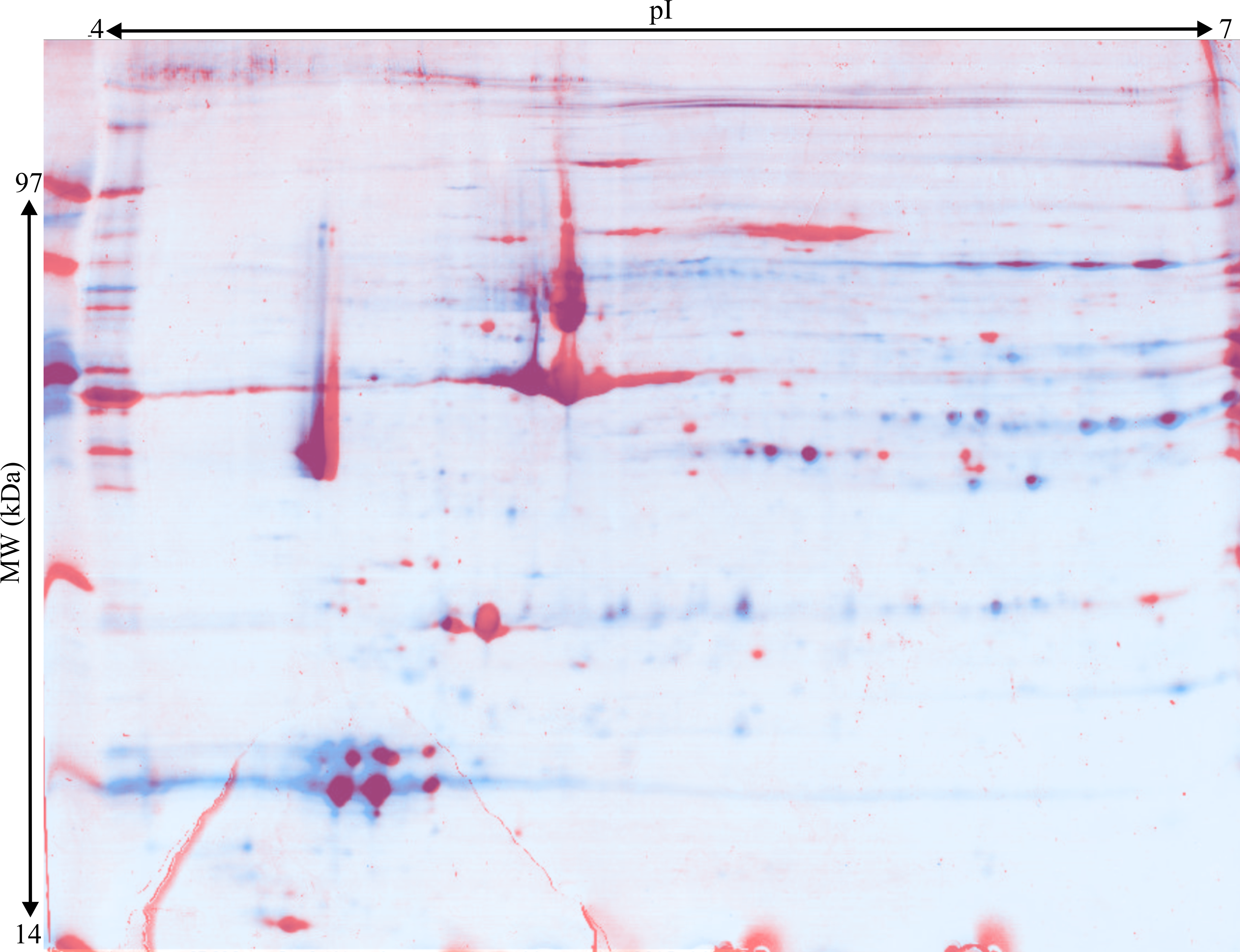

Supplement: S1 Fig — The image obtained with Pro-Q Diamond was colored in blue and the image obtained with Coomassie blue G-250 was colored in red. The overlap of spots in the two images produced brown color. (TIF) [file pone.0170294.s001.tif]

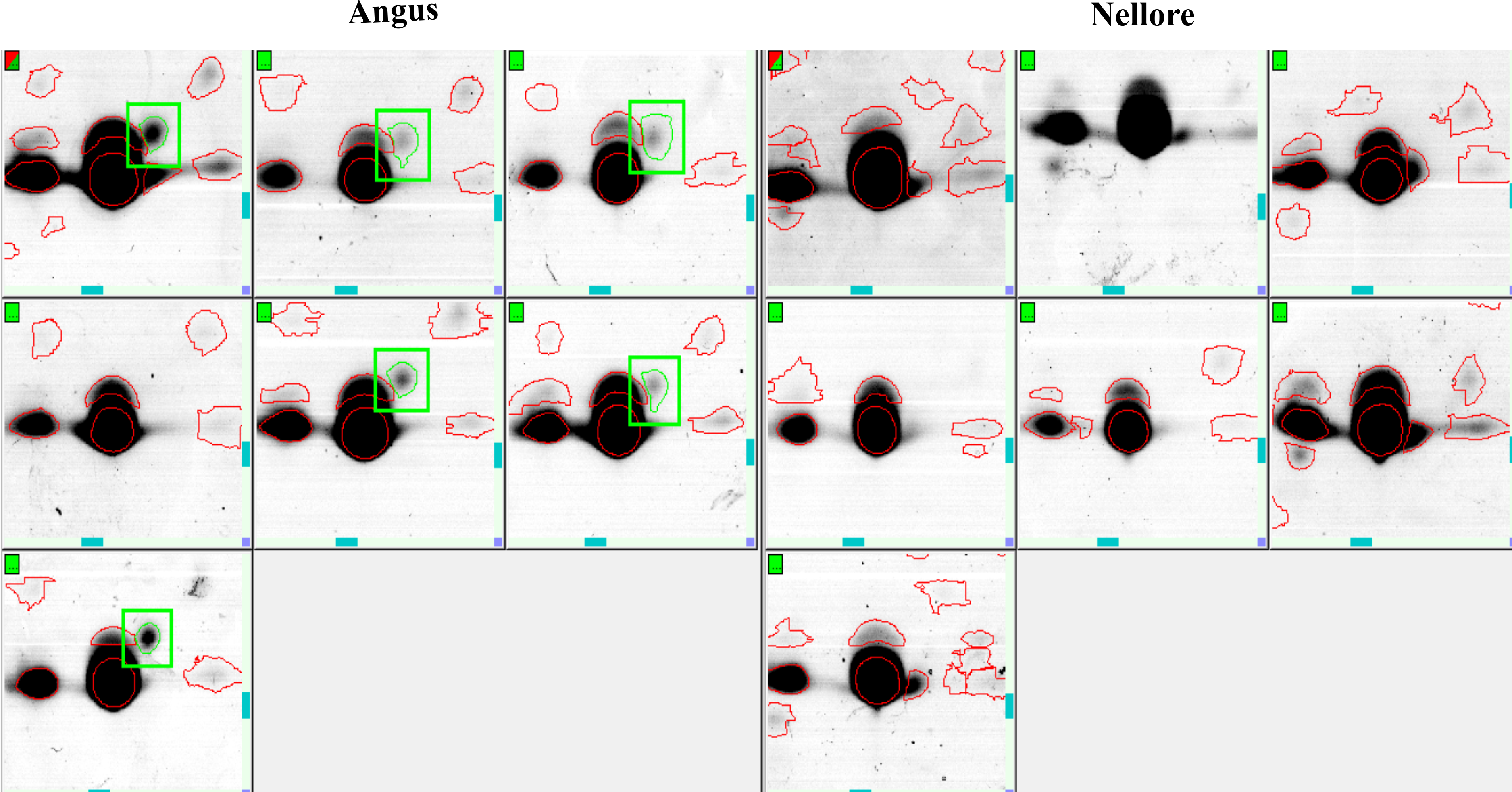

Supplement: S2 Fig — Spots highlighted in the green square (match ID 383) and only detected in Angus muscle. (TIF) [file pone.0170294.s002.tif]

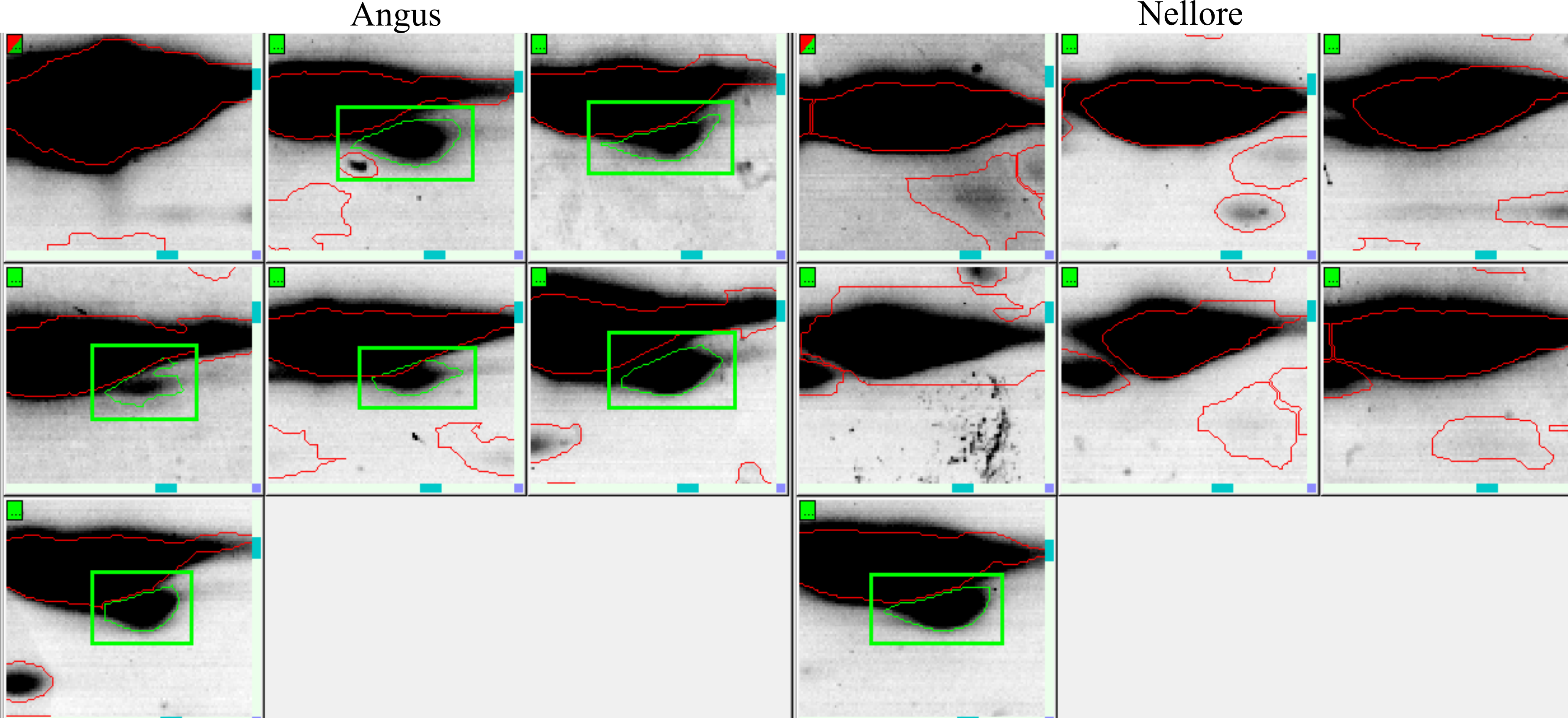

Supplement: S3 Fig — Spots highlighted in the green square (match ID 363) and only detected in the muscle of one of Nellore cattle. (TIF) [file pone.0170294.s003.tif]

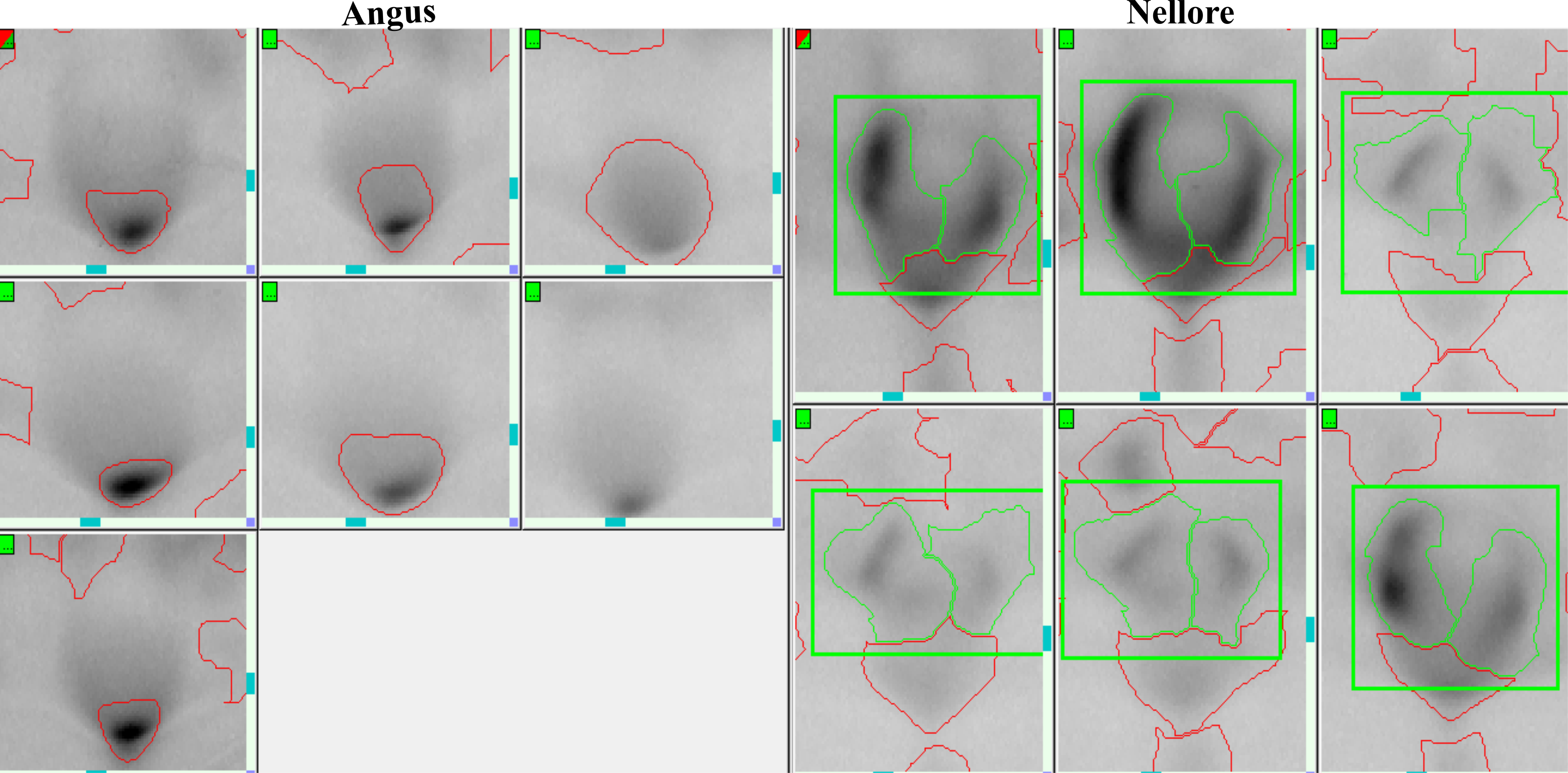

Supplement: S4 Fig — Spots highlighted in the green square (match IDs 1064 and 1061) and only detected in Nellore muscle. (TIF) [file pone.0170294.s004.tif]
