# Supplementary material for: Differences in Beef Quality between Angus (Bos taurus taurus) and Nellore (Bos taurus indicus) Cattle through a Proteomic and Phosphoproteomic Approach
Source: PLoS One. 2017 Jan 19;12(1):e0170294. doi: 10.1371/journal.pone.0170294 (PMC5245812; doi:10.1371/journal.pone.0170294)
Supplement: S1 Table — Sequence of the peptides identified in Mascot and validated by the Scaffold. (DOCX) [file pone.0170294.s005.docx]

S1 Table. Differentially abundant proteins between Angus and Nellore cattle muscle. Sequence of the peptides identified in Mascot and validated by the Scaffold.

| Match ID | Protein ID | Peptide sequence |
| --- | --- | --- |
| More abundant in Angus | | |
| 383 | Myosin light chain 3 | (R)ALGQNPTQAEVLR(V) |
|  |  | (K)DTGTYEDFVEGLR(V) |
| 136 | Phosphoglucomutase-1 | (K)AYQDQKPGTSGLR(K) |
|  |  | (K)LSLcGEESFGTGSDHIR(E) |
|  |  | (K)IDNFEYSDPVDGSISR(N) |
| 199 | Phosphoglucomutase-1 | (K)AYQDQKPGTSGLR(K) |
|  |  | (R)QEATLVVGGDGR(F) |
|  |  | (K)EAIQLIVR(I) |
|  |  | (K)TIEEYAIcPDLHVDLGVLGK(Q) |
|  |  | (R)NIFDFNALK(E) |
|  |  | (R)IDAMHGVVGPYVK(K) |
|  |  | (R)IDAmHGVVGPYVK(K) |
|  |  | (K)TGEHDFGAAFDGDGDR(N) |
|  |  | (K)IALYETPTGWK(F) |
|  |  | (K)LSLcGEESFGTGSDHIR(E) |
|  |  | (K)IDNFEYSDPVDGSISR(N) |
| 106 | Stress-70 protein, mitochondrial | (R)AQFEGIVTDLIR(R) |
| 107 | Stress-70 protein, mitochondrial | (R)AQFEGIVTDLIR(R) |
|  |  | (K)VQQTVQDLFGR(A) |
|  |  | (K)LLGQFTLIGIPPAPR(G) |
| 363 | Uncharacterized protein | (R)IINEPTAAAIAYGLDR(L) |
| 367 | Prohibitin | (R)ILFRPVASQLPR(I) |
|  |  | (R)FDAGELITQR(E) |
|  |  | (R)KLEAAEDIAYQLSR(S) |
| More abundant in Nellore | | |
| 0 | Myosin light chain 1 (fragment) | (K)EAFLLFDR(T) |
|  |  | (K)ITLSQVGDVLR(A) |
|  |  | (K)DQGTYEDFVEGLR(V) |
| 108 | Tropomyosin alpha-1 chain | (K)ATEDELDKYSEALK(D) |
|  |  | (K)KATDAEADVASLNR(R) |
|  |  | (R)RIQLVEEELDR(A) |
|  |  | (R)IQLVEEELDR(A) |
|  |  | (R)KYEEVAR(K) |
|  |  | (R)KLVIIESDLER(A) |
|  |  | (K)SIDDLEDELYAQK(L) |
| 33 | Tropomyosin alpha-1 chain | (R)KLVIIESDLER(A) |
|  |  | (R)RIQLVEEELDR(A) |
|  |  | (R)IQLVEEELDR(A) |
|  |  | (R)KYEEVAR(K) |
|  |  | (K)ATEDELDKYSEALK(D) |
| 48 | Troponin T | (R)KPLNIDHLSEDKLR(D) |
| 29 | Malate dehydrogenase, cytoplasmic | (K)VVEGLPINDFSR(E) |
| 150 | Alpha-enolase | (R)EIFDSR(G) |
|  |  | (R)GNPTVEVDLFTAK(G) |
|  |  | (R)AAVPSGASTGIYEALELR(D) |
|  |  | (K)LAmQEFMILPVGAENFR(E) |
|  |  | (K)LAmQEFmILPVGAENFR(E) |
|  |  | (R)IGAEVYHNLK(N) |
|  |  | (K)DATNVGDEGGFAPNILENK(E) |
|  |  | (K)VVIGmDVAASEFYR(S) |
|  |  | (K)VVIGMDVAASEFYR(S) |
|  |  | (R)YITPDELANLYK(S) |
|  |  | (K)VNQIGSVTESLQAcK(L) |
| 198 | Phosphoglucomutase-1 | (K)AYQDQKPGTSGLR(K) |
|  |  | (R)QEATLVVGGDGR(F) |
|  |  | (K)EAIQLIVR(I) |
|  |  | (R)IDAmHGVVGPYVK(K) |
|  |  | (K)TGEHDFGAAFDGDGDR(N) |
|  |  | (K)IALYETPTGWK(F) |
|  |  | (K)LSLcGEESFGTGSDHIR(E) |
|  |  | (K)IDNFEYSDPVDGSISR(N) |
| 95 | Phosphoglucomutase-1 | (K)TGEHDFGAAFDGDGDR(N) |
| 144 | 78 kDa glucose-regulated protein | (R)ITPSYVAFTPEGER(L) |
|  |  | (K)KSDIDEIVLVGGSTR(I) |
|  |  | (K)SDIDEIVLVGGSTR(I) |
|  |  | (K)DNHLLGTFDLTGIPPAPR(G) |
