# Supplementary material for: Differences in Beef Quality between Angus (Bos taurus taurus) and Nellore (Bos taurus indicus) Cattle through a Proteomic and Phosphoproteomic Approach
Source: PLoS One. 2017 Jan 19;12(1):e0170294. doi: 10.1371/journal.pone.0170294 (PMC5245812; doi:10.1371/journal.pone.0170294)
Supplement: S2 Table — Sequence of the peptides identified in Mascot and validated by the Scaffold. (DOCX) [file pone.0170294.s006.docx]

S2 Table. Differentially abundant phosphoproteins between Angus and Nellore cattle muscle. Sequence of the peptides identified in Mascot and validated by the Scaffold.

| Match ID | Protein ID | Peptide sequence |
| --- | --- | --- |
| More abundant in Angus | | |
| 384 | Troponin T | (K)ALSSmGANYSSYLAK(A) |
|  |  | (R)RKPLNIDHLSEDK(L) |
| 644 | Phosphoglucomutase-1 | (K)AYQDQKPGTSGLR(K) |
|  |  | (K)TGEHDFGAAFDGDGDR(N) |
|  |  | (K)IDNFEYSDPVDGSISR(N) |
| 870 | Phosphoglucomutase-1 | (K)AYQDQKPGTSGLR(K) |
|  |  | (R)QEATLVVGGDGR(F) |
|  |  | (K)EAIQLIVR(I) |
|  |  | (R)IDAMHGVVGPYVK(K) |
|  |  | (R)IDAmHGVVGPYVK(K) |
|  |  | (R)YDYEEVEAEGANK(M) |
| More abundant in Nellore | | |
| 1061 | Myosin light chain 1/3 | (K)QQQDEFKEAFLLFDR(T) |
|  |  | (K)ITLSQVGDVLR(A) |
|  |  | (K)KIEFEQFLPmLQAISNNK(D) |
|  |  | (K)DQGTYEDFVEGLR(V) |
|  |  | (R)HVLATLGEK(M) |
| 1064 | Myosin light chain 1/3 | (R)HVLATLGEK(M) |
| 38 | Myosin regulatory light chain 2 | (K)EAFTVIDQNR(D) |
|  |  | (K)GADPEDVITGAFK(V) |
|  |  | (K)NMWAAFPPDVGGNVDYK(N) |
| 37 | Myosin regulatory light chain 2 | (K)FLEELLTTQcDR(F) |
| 485 | Alpha actin 1 | (R)AVFPSIVGRPR(H) |
|  |  | (K)IWHHTFYNELR(V) |
|  |  | (R)GYSFVTTAER(E) |
|  |  | (K)SYELPDGQVITIGNER(F) |
|  |  | (K)DLYANNVmSGGTTMYPGIADR(M) |
|  |  | (K)QEYDEAGPSIVHR(K) |
| 494 | Alpha actin 1 | (R)AVFPSIVGRPR(H) |
|  |  | (K)IWHHTFYNELR(V) |
| 143 | Triosephosphate isomerase | (K)VPADTEVVcAPPTAYIDFAR(Q) |
|  |  | (K)DLGATWVVLGHSER(R) |
|  |  | (R)HVFGESDELIGQK(V) |
|  |  | (K)TATPQQAQEVHEK(L) |
|  |  | (K)SNVSDAVAQSAR(I) |
|  |  | (R)IIYGGSVTGATcK(E) |
| 179 | 14-3-3 protein épsilon | (K)VAGmDVELTVEER(N) |
|  |  | (K)AASDIAMTELPPTHPIR(L) |
|  |  | (K)AASDIAmTELPPTHPIR(L) |
